# Occult hepatitis C virus infection in Iranian hemodialysis patients

**DOI:** 10.12860/jnp.2015.22

**Published:** 2015-10-01

**Authors:** Ali Eslamifar, Amitis Ramezani, Hassan Ehteram, Effat Razeghi, Farrokhlagha Ahmadi, Manouchehr Amini, Mohammad Banifazl, Gelavizh Etemadi, Hossein Keyvani, Anahita Bavand, Arezoo Aghakhani

**Affiliations:** ^1^Clinical Research Department, Pasteur Institute of Iran, Tehran, Iran; ^2^Department of Pathology, Faculty of Medicine, Kashan University of Medical Sciences, Kashan, Iran; ^3^Nephrology Research Center, Tehran University of Medical Sciences, Tehran, Iran; ^4^Nephrology Department, Tehran University of Medical Sciences, Tehran, Iran; ^5^Iranian Society for Support of Patients with Infectious Diseases, Tehran, Iran; ^6^Chamran Hospital, Tehran, Iran; ^7^Department of Virology, Iran University of Medical Sciences, Tehran, Iran

**Keywords:** Hemodialysis (HD), Hepatitis C virus (HCV), HCV-RNA

## Abstract

*Background:* Occult hepatitis C virus (HCV) infection is defined as the presence of HCV-RNA in liver or peripheral blood mononuclear cells (PBMCs) in the absence of detectable hepatitis C antibody (anti-HCV) or HCV-RNA in the serum. Low concentrations of HCV-RNA may be detected in PBMCs of hemodialysis (HD) patients and this could have a great impact on the management of HD patients.

*Objectives:* The aim of this study was to detect the occult HCV infection in Iranian HD patients.

*Patients and Methods:* A total of 70 anti-HCV negative HD patients from three dialysis units in Tehran, Iran were included in this study. In these cases, presence of HCV-RNA in plasma samples was tested by reverse transcriptase-nested polymerase chain reaction (RT-nested PCR). In cases with negative anti-HCV and plasma HCV-RNA, genomic HCV-RNA was checked in PBMC specimens by RT-nested PCR.

*Results:* Seventy anti-HCV negative HD patients were enrolled in the study. 32.85% and 1.43% of cases had elevated levels of alanine aminotransferase (ALT) and aspartate aminotransferase (AST) respectively. 7.14% of patients had elevated levels of both ALT and AST. HCV-RNA was negative in plasma samples of all anti-HCV negative HD subjects. The genomic HCV-RNA was not detected in any PBMC samples of HD cases with negative anti-HCV and plasma HCV-RNA.

*Conclusions:* Occult HCV infection was not detected in our HD patients despite of elevated levels of liver enzymes in some participants. Further studies involving larger number of HD patients are required to elucidate the rate of occult HCV infection in HD cases.

**Implication for health policy/practice/research/medical education:**

To detect the occult hepatitis C virus (HCV) infection in Iranian hemodialysis (HD) patients, we examined, 70 anti-HCV negative HD patients from three dialysis units. Based on the results of this study, it seems that there is no need for screening of HCV-RNA in plasma and peripheral blood mononuclear cells (PBMCs) of anti-HCV negative hemodialysis subjects.

## 1. Background

Hepatitis C virus (HCV) is a single-strand RNA virus (genomic HCV-RNA) with positive polarity that belongs to the family flaviviridae and genus hepacivirus. Virus replication involves the synthesis of a complementary RNA (antigenomic HCV-RNA) that acts as a template for production of genomic HCV-RNA (1,2). It is estimated that about 170–200 million people (2% of the world’s population) are infected with HCV (3) and its prevalence ranges from 0.2% to 40% in different countries (4,5).

A new entity of HCV infection, occult HCV infection, was first described in 2004 by Castillo et al (6). Occult HCV infection is described as the detection of HCV-RNA in liver or peripheral blood mononuclear cells (PBMCs) in the lack of hepatitis C antibody (anti-HCV) or HCV-RNA in the serum (7). It has been reported that almost 70% of patients with occult HCV infection have HCV-RNA in their PBMCs. Although the most accurate and gold standard method for the diagnosis of occult HCV infection is the detection of HCV genome in the liver, testing of HCV-RNA in PBMCs is an alternative procedure when a liver biopsy is not available (8-10).

Despite screening of blood products for HCV antibody and implementation of universal infection control precautions, HCV infection is still a main health problem in hemodialysis (HD) patients (8). The prevalence of anti-HCV in HD patients is higher than general population, ranging from 7% to 40% (11-13). Low concentrations of HCV-RNA have been detected in PBMCs of patients who were cleared HCV either spontaneously (14,15) or after treatment (16-18). Thus, this could have a great impact on the management of HD patients in dialysis units (19).

## 2. Objectives

To date, only few studies have investigated the presence of occult HCV infection in HD patients (8,20-22). Due to limited data regarding this issue, especially in Iranian HD patients, this study was performed to determine the existence of occult HCV infection in Iranian HD cases.

## 3. Patients and Methods

In this cross-sectional study, 70 anti-HCV negative HD patients were recruited from three dialysis units in Tehran, Iran from March to September 2013. A questionnaire was used to collect data such as age, sex and length of time on dialysis.

All enrolled patients were negative for anti-HCV by 2 enzyme-linked immunosorbent assays (ELISA) (Biorad, Segrate, Italy).

All of the cases were also negative for hepatitis B surface antigen (HBsAg) and anti-HIV antibodies.

Liver enzymes (alanin aminotransferase [ALT] and aspartate aminotransferase [AST]) were also determined in all of the patients using the Hitachi 704 autoanalyser, Tokyo, Japan. The ALT levels above 17 IU/l and the AST levels above 24 IU/l were considered as abnormal in HD patients (23).

A peripheral blood sample from each patient was collected in an EDTA-containing sterile tube. Plasma was separated by centrifugation and stored at –80°C for further tests.

The PBMCs were isolated by Ficoll Hypaque (FH) gradient centrifugation (Lympholyte-H, Cedarlane, Canada). The PBMCs were washed 3 times with phosphate-buffered saline (PH = 7.3 ± 0.1). The cells were counted and after adding RNAlater (Ambion Inc., Austin, TX) solution, were stored at –80°C until examination. Plasma and PBMC specimens from 10 patients with chronic HCV infection and 10 blood donors were used as positive and negative controls, respectively.

### 3.1. Reverse transcriptase-nested polymerase chain reaction (RT-nested PCR)

RNA was extracted from plasma and a pellet of about 3-5×10^6^ PBMC specimens using High Pure Viral Nucleic Acid Kit (Roche Diagnostics GmbH, Mannheim, Germany) according to the manufacturer’s instructions.

Genomic HCV-RNA in plasma and PBMC specimens was detected by RT-nested PCR method. The cDNA synthesis and a 2-step PCR with nested primers were performed as described previously (24-26). The PCR products were electrophoresed in a 1.5% agarose gel and stained with ethidium bromide.

The sensitivity of the PCR amplification method for detection of genomic HCV-RNA was 40 IU/ml plasma. The sensitivity was determined by testing serial dilutions of a plasma specimen with a known viral load (determined using Cobas TaqMan 48 Analyzer [Roche, Germany]) (26).

### 3.2. Ethical issues

1) The research followed the tenets of the Declaration of Helsinki; 2) This project was approved by the Iranian Society for Support of Patients with Infectious Diseases ethics committee (No. 87) and informed consent was obtained from patients prior to their enrollment.

### 3.3. Statistical analysis

The SPSS 16 package program (Chicago, IL, USA) was used for data analysis. Data are presented as mean ± SD or, when indicated, as an absolute number and percentage.

## 4. Results

A total of 70 anti-HCV negative HD patients with mean age 58.9 ± 14.7 (range: 24-89) years were enrolled in the study. 45.7% of patients were male and 54.3% were female. The mean duration of HD was 5.9 ± 4.9 years. The mean ALT and AST levels were 17.7 ± 9.5 (range: 8-71) and 19.1 ± 8.1 (range: 8-56) IU/l respectively. 32.85% (23) and 1.43% (1) of cases had elevated levels of ALT and AST respectively. Five (7.14%) patients had elevated levels of both ALT and AST.

HCV-RNA was negative in plasma samples of all anti-HCV negative HD patients. The genomic HCV-RNA was not detected in any PBMC samples of HD cases with negative anti-HCV and plasma HCV-RNA. It means that there was no occult HCV infection in our HD subjects.

## 5. Discussion

In this study the presence of occult HCV infection in Iranian HD patients was determined. The genomic HCV-RNA was not detected in PBMCs of these cases. Our survey showed that occult HCV infection was not found in Iranian HD patients despite of sex, age, length of time on dialysis and aminotransferases levels.

HCV infection is a major cause of chronic liver disease and it is diagnosed by the detection of anti-HCV and/or HCV-RNA in serum. In the past years, a new entity of HCV infection was identified and defined as occult HCV infection, in which HCV-RNA is undetectable in serum by conventional assays, but patients have HCV-RNA in liver or PBMCs (6,27). Occult HCV infection is distributed throughout the world and all HCV genotypes are involved in this infection. Occult HCV infection has been found in different high-risk groups for HCV infection such as HD cases and family members of occult HCV patients. It seems that this infection is less aggressive than chronic HCV infection although patients with occult HCV may develop liver cirrhosis and hepatocellular carcinoma (7).

Occult HCV infection has been previously investigated in HD patients in few studies. In a survey by Barril et al (8) occult HCV infection was found in 45% of HD patients with abnormal liver enzyme levels in Spain. These patients had a significantly longer time on HD. Thongsawat et al (21) investigated occult HCV infection during an outbreak in a HD unit in Thailand and reported that occult HCV infection was common among HD patients in this country. Rinonce et al (22) showed that occult HCV infection was detectable in 12.9% of HD patients in Indonesia. They suggested that implementation of strict infection control programs is necessary in HD units in this country. Yakaryilmaz et al (20) reported that the prevalence of occult HCV infection in Turkish HD patients was 4.8%.

These differences between the rates of occult HCV infection in dialysis patients may be due to diverse prevalence of HCV infection in different countries and within different dialysis units. The overall seroprevalence of HCV infection in the general population of Iran is low and about 0.5% (1.0 in men and 0.1 in women). The prevalence of anti-HCV in HD patients is higher than general population, ranging from 11%-25% (28). Routine serologic screening of HCV infection, together with strict adherence to universal precautions cause to control HCV infection in HD patients and decreasing the rate of HCV infection in HD cases (28,29). The overall rate of HCV infection in our three studding HD units was about 6%; so it is not unexpected that occult HCV infection was rare in our dialysis patients.

As mentioned before, although the detection of HCV-RNA in the liver biopsy specimen is the gold standard method for diagnosis of occult HCV infection, detection of HCV-RNA in PBMCs is an alternative procedure in the absence of liver biopsy (8,21) and HCV-RNA was detected in the PBMCs of 70% of patients with occult HCV infection (6,8). Therefore detection of HCV-RNA in PBMCs does not identify all cases with occult HCV and because of liver biopsy is not routinely recommended for HD patients, some of the HD cases without HCV-RNA in their PBMCs could have occult HCV infection in their liver. Therefore, it is possible that the rate of occult HCV infection in our HD patients was actually higher than those reported in this study.

Occult HCV infection has been described in two different clinical settings: in patients with normal liver enzymes (17,18), or in patients with abnormal values of liver enzymes (6,25). In this study we found no occult HCV infection in our cases despite of increased levels of liver enzymes in some subjects.

Some scholars (8,30) reported that duration of HD was significantly longer in patients with occult HCV infection, but we did not find this state in our patients.

## 6. Conclusions

Occult HCV infection was not detected in our HD patients despite of elevated levels of liver enzymes in some participants. Further studies involving larger number of HD patients are required to elucidate the rate of occult HCV infection in HD cases.

## 7. Limitations of study

The limitation of our study is the relatively small sample size. Therefore, further studies with more cases are needed to elucidate the rate of occult HCV infection in HD patients.

## Acknowledgements

The authors are grateful to Iranian society for support of patients with infectious diseases.

## Authors’ contribution

Study concept and design: AR and AA. Acquisition of data and sampling: FA, ER, MA, HK, and AB. Analysis and interpretation of data: AE and HE. Drafting of the manuscript: AA. Critical revision of the manuscript for important intellectual content: AR and AA. Statistical analysis: GE. Study supervision: AA, AR, and MB.

## Conflicts of interest

The authors declare no conflict of interest.

## Funding/Support

This project was financially supported Iranian society for support of patients with infectious disease (grant No. 87).
